# Fronto-Parietal Contributions to Phonological Processes in Successful Artificial Grammar Learning

**DOI:** 10.3389/fnhum.2016.00551

**Published:** 2016-11-08

**Authors:** Dariya Goranskaya, Jens Kreitewolf, Jutta L. Mueller, Angela D. Friederici, Gesa Hartwigsen

**Affiliations:** ^1^Department of Neuropsychology, Max Planck Institute for Human Cognitive and Brain SciencesLeipzig, Germany; ^2^International Laboratory for Brain, Music and Sound Research, Department of Psychology, University of Montreal, MontrealQC, Canada; ^3^Institute of Cognitive Science, University of OsnabrückOsnabrück, Germany

**Keywords:** functional magnetic resonance imaging, premotor cortex, parietal cortex, phonological processes, phonological segmentation, phoneme comparison, auditory sequence processing, learning

## Abstract

Sensitivity to regularities plays a crucial role in the acquisition of various linguistic features from spoken language input. Artificial grammar learning paradigms explore pattern recognition abilities in a set of structured sequences (i.e., of syllables or letters). In the present study, we investigated the functional underpinnings of learning phonological regularities in auditorily presented syllable sequences. While previous neuroimaging studies either focused on functional differences between the processing of correct vs. incorrect sequences or between different levels of sequence complexity, here the focus is on the neural foundation of the actual learning success. During functional magnetic resonance imaging (fMRI), participants were exposed to a set of syllable sequences with an underlying phonological rule system, known to ensure performance differences between participants. We expected that successful learning and rule application would require phonological segmentation and phoneme comparison. As an outcome of four alternating learning and test fMRI sessions, participants split into successful learners and non-learners. Relative to non-learners, successful learners showed increased task-related activity in a fronto-parietal network of brain areas encompassing the left lateral premotor cortex as well as bilateral superior and inferior parietal cortices during both learning and rule application. These areas were previously associated with phonological segmentation, phoneme comparison, and verbal working memory. Based on these activity patterns and the phonological strategies for rule acquisition and application, we argue that successful learning and processing of complex phonological rules in our paradigm is mediated via a fronto-parietal network for phonological processes.

## Introduction

Successful speech processing and language learning rests on efficient processing of sequential auditory information and establishing relationships between speech elements (Hasson and Tremblay, 2015). Different elements of speech, such as phonemes, syllables, or words, are sequentially organized into speech streams, following language-specific constraints (or rules). Converging evidence from genetic, non-human primate and cognitive neuroscience studies indicates that language and sequence learning have considerable overlap in the underlying neural mechanisms. It was thus argued that sequence learning mechanisms are important in language acquisition (Christiansen and Chater, 2015).

Artificial grammar (AG) learning paradigms provide a means to study sequence learning during language acquisition and its evolution both in human populations (children and adults) and in non-human species (e.g., primates or birds; Fitch and Friederici, 2012). One major advantage of AG learning paradigms relative to the study of natural language stimuli is that they allow for exploring the processing of rule-based regularities in a controlled way and unconfounded by semantic processes and prior knowledge (Fitch and Friederici, 2012). These paradigms usually include a learning phase and a test phase. During learning, participants are exposed to a set of syllable or letter sequences and during testing, their abilities to detect the underlying rule system are assessed (Lobina, 2014). In the test phase, participants are usually presented with novel sequences and need to classify them as correct or incorrect according to the underlying rules acquired in the learning phase (cf. Bahlmann et al., 2008). From the language acquisition perspective, AG learning represents one of a few language acquisition paradigms suitable for adult subjects. In previous studies, AG learning paradigms were mainly used as a model for syntactical and phonological processing (Uddén, 2012).

Previous neuroimaging, electrophysiological and behavioral studies used AG learning paradigms to elucidate the human capacity to detect regularities in speech sequences. Specifically, these studies investigated the processing of structured syllable sequences in healthy participants (Friederici et al., 2006; Bahlmann et al., 2008; Mueller et al., 2012; Uddén and Bahlmann, 2012). Some of the previous AG learning studies used shared articulatory features as cues for the underlying structure of the presented sequence (e.g., sequences like “di-ge-ku-to,” whereby place of articulation indicates which syllable pairs belong together, namely “di-to” and “ge-ku”; Bahlmann et al., 2008; Mueller et al., 2010). It was argued that during successful AG learning in these studies, participants have to realize the pairing rule between syllables, specifically, between their starting consonants (Lobina, 2014). As soon as the relevant consonant pairs are linked by phonetic features, these features need to be rehearsed in phonological working memory to link up the different pairs.

Also, previous electrophysiological studies addressed the contribution of specific cognitive processes to language learning. These studies suggest that in the absence of semantic information during early stages of language learning, as with AG learning paradigms, attention and working memory processes are critical (De Diego-Balaguer and Lopez-Barroso, 2010).

Yet, a number of distinguishable processes are likely to contribute to successful learning of AG structures. Basic cognitive processes that are likely involved in learning include: (1) sensory or input encoding; (2) pattern extraction; (3) model building; and (4) retrieval or recognition processes (Karuza et al., 2014). Presumably, the relative involvement of these processes (and their interaction) changes in the course of a learning task. For instance, in the early phase of learning model building processes should closely interact with pattern extraction processes. In the later stages one would expect interaction of model building and recognition/retrieval processes to become more prominent. Moreover, the retrieval or recognition processes are likely to be necessary in the assessment procedure, when the outcome of learning is measured and participants need to demonstrate acquired knowledge (Karuza et al., 2014).

While most of the previous studies on AG learning focused on the outcome of learning by testing acquired rule knowledge (Friederici et al., 2006; Bahlmann et al., 2008, 2009), the neural correlates of the successful rule acquisition process itself remain largely unclear with some exceptions (Opitz and Friederici, 2004). Most studies tested processing of the AG sequences after learning and not during learning, which is likely to draw on the rule retrieval processes in the above mentioned learning framework (Karuza et al., 2014). We aimed at unraveling the functional underpinnings of successful auditory learning of phonological rules in AG sequences, focusing on the processes of pattern extraction and model building. To this end, we relied on a complex AG learning paradigm that was previously used in a behavioral study which showed reliable learning effects but also a large degree of variation in learning success (Mueller et al., 2010). In the present study, we used the known difficulty of these structures as a means to obtain variance in learning success, as we focused on the comparison between successful and unsuccessful rule learning processes. The stimuli were unconfounded by any semantic information and should thus allow for the investigation of “pure” phonological learning processes. A better understanding of the processes underlying rule extraction in AG learning paradigms might help to establish valid functional-anatomical models of rule acquisition in the healthy brain.

With respect to the underlying network for AG learning, some neuroimaging studies observed (left-lateralized or bilateral) fronto-parietal areas, including the left dorsal and ventral premotor cortex (PMC) and left inferior and superior parietal cortex (Fletcher et al., 1999; Tettamanti et al., 2002). Other studies (Friederici et al., 2006; Bahlmann et al., 2008, 2009) reported increased task-related activation in the left posterior inferior frontal gyrus (pIFG, pars opercularis) for the processing of sequences with complex as compared to simple structures and for violations of the sequence structure.

Based on the above described contribution of phonological processes to AG rule learning, we hypothesized that a successful strategy in rule learning should involve phonological processes, including phonological working memory for silent rehearsal. Specifically, successful AG learning and rule application should include search processes for specific phonetic features (i.e., consonants), phonological segmentation processes and phoneme discrimination for phoneme extraction, as well as silent rehearsal and phoneme comparison among long-distance elements. These processes should engage a left-dominant fronto-parietal network that was previously associated with phonological processes (e.g., Baddeley, 1992). If our task engages speech segmentation and phonological working memory processes, we would expect increased task-related activity in the left lateral PMC and adjacent pIFG (e.g., Paulesu et al., 1993; Cunillera et al., 2009; Price, 2012), since this region should contribute to the active maintenance of non-meaningful verbal representations through articulatory subvocal rehearsal (Smith and Jonides, 1998) and might be crucially engaged in the first stages of learning unfamiliar new words (Baddeley et al., 1998). Additionally, we aimed to test whether the posterior IFG, which has been shown previously to be sensitive to the processing of complex AGs, would be also involved in initial stages of learning. Moreover, a posterior region in the inferior/superior parietal cortex should also contribute to phonological working memory (e.g., Kirschen et al., 2006; Romero et al., 2006), particularly to the short-term storage of information (see Buchsbaum and D’Esposito, 2008 for review). Both parietal and premotor areas were assigned to the dorsal language stream (Hickok and Poeppel, 2004, 2007) that comes into play when it is necessary to keep auditory representations in an active state during task performance (Buchsbaum et al., 2005; Aboitiz et al., 2006; Jacquemot and Scott, 2006) and contributes to novel word or phoneme learning (Rodriguez-Fornells et al., 2009).

## Materials and Methods

### Participants

Initially, 65 healthy young volunteers (31 females) participated in the study. Four subjects had to be excluded due to technical reasons (data quality or missing behavioral responses). All of the remaining participants (31 males, 30 females; age range 20–36 years, mean age ± SD: 26.9 ± 3.81 years) were right-handed according to the Edinburgh handedness questionnaire (Oldfield, 1971). They were native German speakers with hearing levels within normal limits. Hearing levels were tested in octave steps between 250 and 8000 Hz in both ears using pure-tone audiometry. Normal hearing limits were defined as a maximum of 20 dB. They had no history of neurological or psychiatric disorders, no drug or alcohol abuse, no current pregnancy, no chronic medical disease, and no contraindications to MR-scanning. Written informed consent was collected from all participants according to the procedures approved by the local Ethics Committee of the University of Leipzig. Participants were paid after completing the experiment.

### Stimuli

**FIGURE 1:**
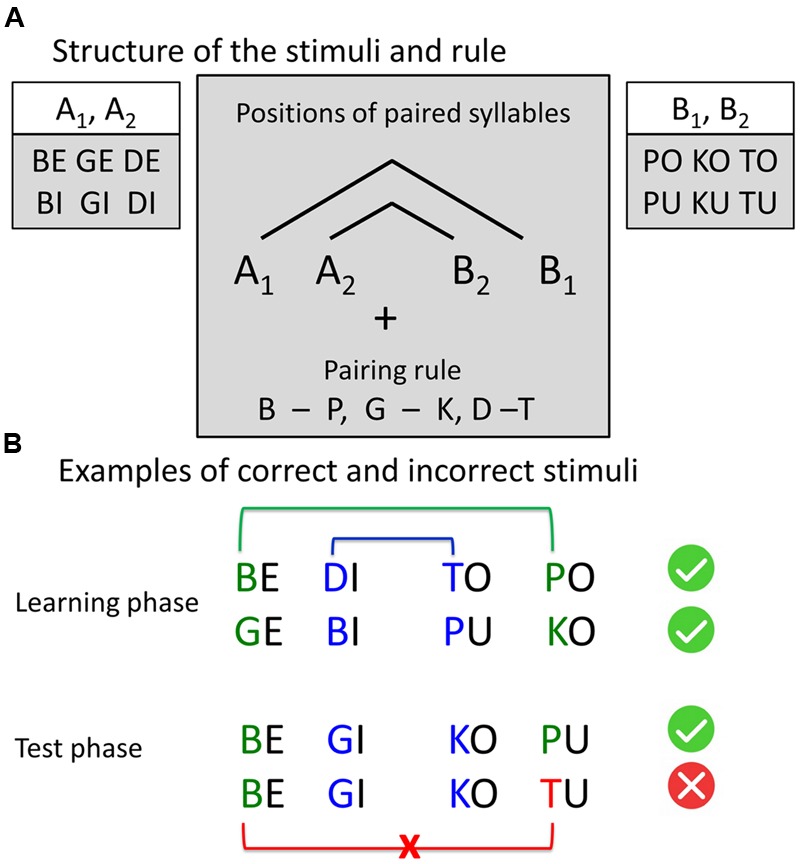
**Stimuli structure and examples. (A)** Structure of the stimuli and the rule. Four-syllable sequences were constructed according to the artificial grammar rule A_1_A_2_B_2_B_1_, where A and B represent two classes of consonant-vowel syllables and indices 1 and 2 represent the pairing rule. The consonants, sharing the same place of articulation (pairs “b” – “p,” “g – k,” “d – t”), were paired by a center-embedding rule: The first consonant was paired with the last consonant, and the second consonant was paired with the third consonant. All sequences, following this rule, were correct. In incorrect sequences, the last consonant violates the pairing rule. **(B)** Examples of correct and incorrect stimuli.

We used naturally spoken four-syllable sequences built according to AG rules with center-embedded structure (**Figure 1**) described in previous studies (e.g., Mueller et al., 2010). There were twelve different consonant-vowel syllables. Our AG had the structure A_1_A_2_B_2_B_1_ with pairwise dependencies between consonants of the syllables of two classes: A and B. Class A included syllables with voiced plosives and front vowels “e” and “i”: {be, bi, ge, gi, de, di}, class B included syllables with voiceless plosives and back vowels “o” and “u”: {po, pu, ko, ku, to, tu}. A and B refer to the vowel component, while indices 1 and 2 refer to the consonant component of the syllable (e.g., “be-gi-ko-pu,” “be-de-tu-po”). To increase the saliency of the dependency, paired consonants (d-t, g-k, b-p) were phonetically similar (shared place of articulation). In the sequence “be-gi-ko-pu” place of articulation indicates which syllable pairs belong together, namely “be-pu” and “gi-ko”. This led to a total set of 96 different four-syllable sequences, built according to the grammar rules (correct items) and 32 different four-syllable sequences with rule violations (incorrect items). Incorrect items had a violation of congruency between the first and the fourth element of the sequence (e.g., A_1_A_2_B_2_B_3_ sequences, like “be-gi-ku-to”). Similar sequences were previously employed to assess the learnability of complex, embedded (“syntactic”) structures by using phonological cues in the input signal (Mueller et al., 2010).

### Experimental Design and Procedures

#### Pre-scanning Training

Before the experiment, participants were familiarized with the structure and the presentation rate of the stimuli. To avoid that participants could acquire knowledge about the structure of the four-syllable sequence, we used spectrally rotated versions of the syllables (Blesser, 1972). This ensured unintelligibility, while maintaining the duration of the syllables. During presentation of each four-syllable sequence, the word “Listen” was presented on a computer screen. During the silence period between two sequences, the word “Respond” was presented and participants were instructed to press any response key (corresponding to “yes” or “no”) before the presentation of the next sequence. The inter-trial interval was identical to that of the main experiment (i.e., 2.5 s). The aim of this familiarization procedure was to reduce the number of missing responses in the main experiment.

We also included a short familiarization session that required the participants to passively listen to two-syllable sequences A_j_B_j_ that followed the same rule as the four-syllable sequences used in the main experiment. The practice session consisted of 12 two-syllable sequences, repeated three times in randomized order to facilitate learning (Mueller et al., 2010).

#### Scanning Sessions

**FIGURE 2:**
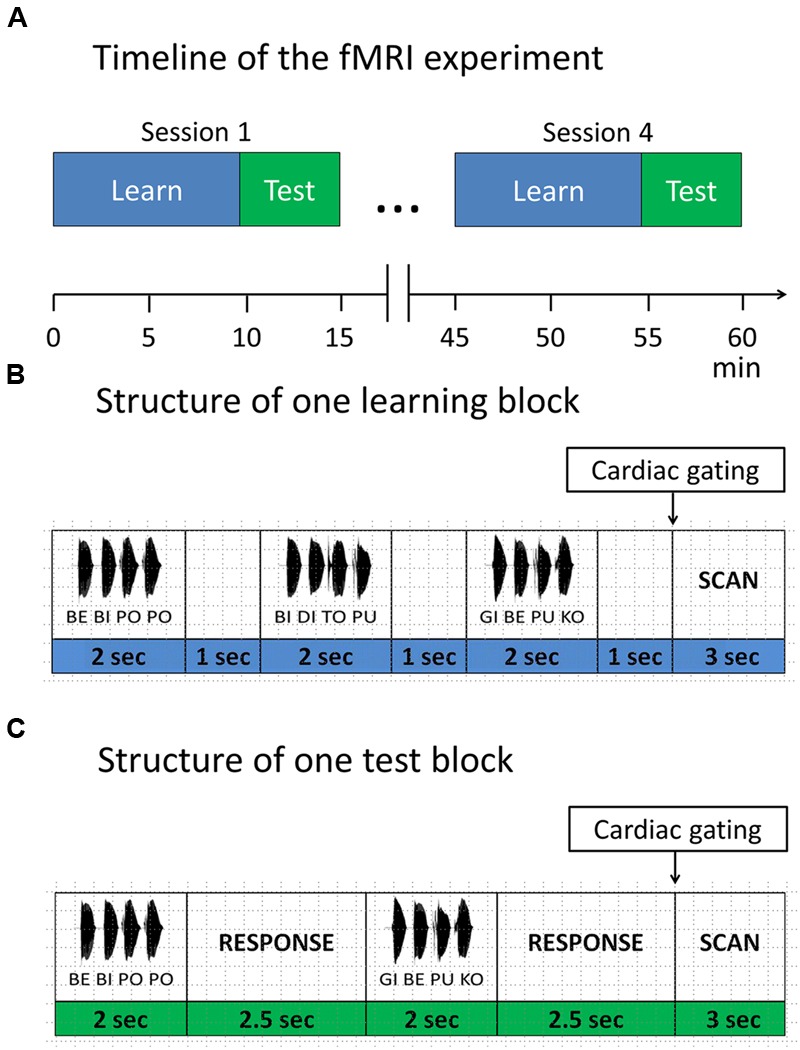
**Functional magnetic resonance imaging (fMRI) paradigm. (A)** Timeline of the fMRI experiment. The experiment consisted of four sessions. Each session started with a learning phase, followed by a test phase. Items were presented in blocks. **(B)** Structure of one learning block. Each learning block consisted of three items, separated by 1 s. **(C)** Structure of one test block. Each test block consisted of two items, separated by a response window of 2.5 s. After each test item participants responded with a button press, whether the test item was correct with respect to the rule or not.

In the main experiment participants performed four scanning sessions of a block design, each lasting approximately 15 min, with 9 min learning phase and 5 min test phase (**Figure 2**). Each session included two short visual instructions, indicating the start of the corresponding phase (i.e., learning or test phase, **Figure 2**). Participants were informed that the same rule was used in all learning phases and that all included items in learning phases were correct, while in the test phases correct and incorrect items were presented. To avoid interference with the auditorily presented stimuli, MRI volumes were acquired after each block in an interleaved fashion (see “Magnetic Resonance Imaging section” for details). During the whole procedure a fixation cross was displayed.

In the learning phase, subjects were instructed to listen attentively and try to detect any regularities in the speech stream. Each learning phase comprised of 32 learning blocks. In each block, three four-syllable sequences were presented with an interstimulus interval of 1 s and a short pause between sequences, leading to a total number of 96 different correct sequences per session, presented in a randomized order.

In the test phase, participants performed a discrimination task requiring them to judge whether a given sequence was correct with respect to the rule they had previously learned. They were instructed to guess in case they didn’t know the rule yet. In each of the 16 test blocks, two four-syllable sequences were presented with an interstimulus interval of 2.5 s, leading to a total of 32 test sequences per session (i.e., 16 correct sequences vs. 16 incorrect sequences).

After each test item, participants had to indicate via button press whether the current sequence followed the previously learned rule (binary answers: yes vs. no). Responses were collected within a time window of 4.5 s from the stimulus onset. We also included null blocks (i.e., 16 null blocks in each learning session and 8 null blocks in each test session) to avoid habituation. Participants were instructed to respond with the index and middle finger of their right hand on a custom-made two-button response box. Presentation of stimuli, recording of participants’ responses and synchronization of the experiment with the MR scanner was accomplished using Cogent 2000^1^.

#### Post-scanning Assessment

After the functional magnetic resonance imaging (fMRI) experiment, participants went through a structured interview that assessed individual strategies during rule acquisition and application as well as rule knowledge. It also covered hypotheses about the rule, attention focus (whole syllables sequences, isolated syllables, vowels, consonants) as well as application and modification of rules in the test phases. Participants were further asked to generate some examples of the correct syllable sequences, which followed the rule they were learning. General questions addressed clear stimulus presentation, motivation, fatigue, and attention drops during different phases of the experiment. Thereafter, participants underwent a short post-scanning test that required them to judge 11 test items to confirm presence or absence of rule knowledge. They were asked to verbally explain their decisions and, in the presence of rule knowledge, explicitly show how they would apply the rule on each of the test items, which included both correct and incorrect examples.

### Magnetic Resonance Imaging

MRI data were obtained using a 3T Siemens Tim Trio MR scanner (Siemens Medical Systems, Erlangen, Germany). Auditory stimuli were delivered using MR-compatible headphones (MR confon GmbH, Magdeburg, Germany). To attenuate scanner noise, participants wore flat frequency-response earplugs (ER20; Etymotic Research, Inc., Elk Grove Village, IL, USA). Prior to fMRI, sound levels were individually adjusted to a comfortable hearing level for each participant.

To avoid masking of the auditory stimuli by scanner noise, we used sparse temporal sampling (Hall et al., 2000; Gaab et al., 2007). Gradient-echo planar images (EPIs) were acquired in an interleaved fashion after each presentation block (whole brain coverage, 42 transverse slices in ascending order per volume; flip angle, 90°; acquisition bandwidth, 116 kHz; TR: 12 s; TE: 30 ms; TA: 2730 ms; matrix size 192 × 192; 2 mm slice thickness; 1 mm interslice gap; in-plane resolution, 3 × 3 mm; cardiac triggering). Additionally, cardiac gating was applied. On each block, after 9 s had elapsed, the scanner waited for the first heartbeat to trigger volume acquisition. Due to this, the actual repetition time (TR) was variable (mean: 12.49 s; *SD*: 365 ms, across all participants). In total, 300 brain volumes were acquired in four scanning sessions for each participant. Task instructions and fixation cross during auditory presentations were delivered using a LCD projector (PLC-XP50L, SANYO, Tokyo, Japan), which could be viewed via a mirror located above the head coil.

High-resolution T1-weighted MR scans of each participant were taken from the in-house database (standard MPRAGE sequences, whole brain coverage, voxel size 1 mm isotropic, matrix size 240^∗^256^∗^176, TR: 2300 msec, TE: 2.98 msec, TA: 6.7 min, sagittal orientation, flip angle 9°).

### Statistical Analyses

#### Behavioral Data

For analyses of reaction times (RTs), we first excluded incorrect responses and misses from further analysis and performed outlier correction on the individual level (i.e., by excluding trials with response speed deviating more than two SD from the individual mean within each subject). Note that this resulted in an imbalance of the total number of items included for learners and non-learners. RTs were measured from the onset of the test item until button press within a time window of 4.5 s.

#### fMRI data

Functional images of fMRI sessions were analyzed with Statistical Parametric Mapping software (SPM12; Wellcome Trust Centre for Neuroimaging^2^; Friston et al., 2007) implemented in Matlab (release 2015). Standard preprocessing procedures comprised correction of motion-related artifacts (realignment and unwarping), coregistration of the T1 and mean EPI images in each participant, segmentation, normalization into standard stereotactic space [Montreal Neurological Institute (MNI) template], and spatial smoothing with a Gaussian kernel of 8 mm FWHM, and high-pass filtering at 128 s (Friston et al., 2007).

On the individual first level, statistical parametric maps (SPMs) were generated by modeling the evoked hemodynamic response for the two conditions (i.e., learning and test) as boxcar functions convolved with a canonical double gamma hemodynamic response function within the general linear model (Friston et al., 2007), with the null blocks forming an implicit baseline. All four runs were assigned an equal weight. T-contrasts were computed for the main effect of each condition (learning and test) and for the contrast learn + test > implicit baseline.

At the second-level, between-group analyses (learners > non-learners and non-learners vs. learners) were performed with two-sample *t*-tests on the individual contrast images (learn + test > baseline, learn > baseline, test > baseline). Results were thresholded at a cluster level of *p* < 0.05, FWE corrected using an initial threshold of *p* < 0.001 (uncorrected) and a cluster threshold of *k* > 250 voxels (Friston et al., 1994).

Effect sizes for regions of interest (ROIs) were calculated as percent signal change with the rfxplot toolbox (Gläscher, 2009). ROIs were defined individually as a sphere with 3-mm radius centered on the individual subject peak, located in the vicinity of the corresponding group peak (within 6 mm) for the two main clusters from the contrast (learn + test > baseline). Neuroimaging data was visualized with MRIcron^3^.

## Results

### Subject Classification: Learners vs. Non-learners

Based on the outcome of the post-scanning assessment, participants were classified as either learners or non-learners. Participants were only classified as learners if they were able to explicitly explain the full rule (as presented in **Figure 1**) and apply it correctly to all items of the post-scanning test (i.e., 100% correct answers in classifying the examples). Learners explained if the test items were correct or incorrect according to the full rule and explicitly indicated the position of violation and the violating consonant. With respect to the learned rule, all learners reported the four-syllable structure of the sequences. They identified two parts of the syllable sequence, with vowels “e,” “i” and consonants “b,” “d,” “g” in the first two-syllables and vowels “o,” “u” and consonants “p,” “t,” “k” in the last two-syllables. They noted that the second and third syllable, as well as first and forth syllable were paired, leading to a pairing of “similar” consonants such as “b” and “p,” “d” and “t,” “g” and “k.” They also reported that the structure of the last two consonants mirrored the sequence of the first two consonants in reversed order.

**FIGURE 3:**
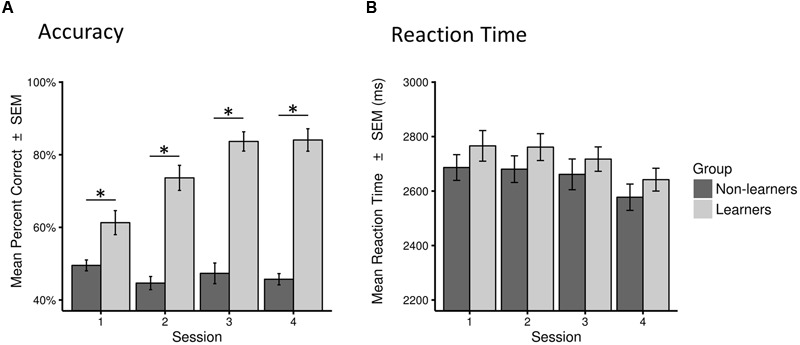
**Behavioral performance (accuracy and reaction time) for learners and non-learners across sessions. (A)** Accuracy as percentage of correct answers across all trials in each session (mean percent correct ± SEM). **(B)** Reaction time across all correct trials in each session in milliseconds (mean ± SEM). Statistical significance (*p* < 0.01) is marked by ^∗^.

This classification led to a final sample of 30 learners (mean age 26.1 years; 19 females) and 31 non-learners (mean age 27.65 years; 11 females). All learners, except two, showed accuracy above 72% across test sessions. Learning success was also reflected in performance increase across test sessions, as shown in **Figure 3** (see also **Supplementary Figure S1**).

There were no between-group difference in mean RTs in any of the four sessions (Mann–Whitney test, all *W* > 373, *p* > 0.05). However, as expected, learners performed significantly better than non-learners in all sessions (*W* = 282.5, *p* < 0.01 in the first session; *W* = 84.5, 52.5, 65.0, *p* < 0.001 in all other sessions; surviving a Bonferroni–Holm correction for multiple comparisons; **Table 1**; **Figure 3**, **Supplementary Figure S1**).

**Table 1:** Behavioral data for learners and non-learners.

	Session 1	Session 2	Session 3	Session 4
**Mean accuracy (%) + SEM**				
Learners	62% ± 3.4%	74% ± 3.5%	84% ± 2.7%	84% ± 3.2%
Non-learners	50% ± 1.5%	45% ± 1.9%	47% ± 2.9%	46% ± 1.6%
Effect size (Cohen’s d)	0.83	1.87	2.40	3.11
**Mean reaction time (ms) + SEM**				
Learners	2765.93 ± 56.22	2761.40 ± 49.24	2717.38 ± 44.78	2641.90 ± 41.89
Non-learners	2686.46 ± 47.21	2680.45 ± 78.91	2661.36 ± 56.39	2577.43 ± 48.31
Effect size (Cohen’s d)	0.28	0.22	0.20	0.46

### Behavioral Strategies

In the post-scanning assessment learners reported two main strategies that were used both during learning and in the test phase after successful rule learning: a forward prediction strategy and a simple strategy. The two strategies were reported in a free manner, based on introspection (in response to the question: “Which strategies did you use during learning and test phase, respectively?”). We did not perform any quantification of response strategies since the same subject could use different strategies throughout the experiment. Notably, all learners explicitly reported the four-syllable structure of the sequences (see subject classification above).

With respect to the reported learning strategies, it should be borne in mind that our stimuli consisted of pairs of voiced and voiceless plosives between syllables 2 and 3 (“inner pair”) and syllables 1 and 4 (“outer pair”) (i.e., “b” was paired with “p,” “d” with “t” and “g” with “k,” **Figure 1**). For successful rule application, a learner needed to confirm that this pairing was present in the correct item or violated in the incorrect item. This was verbally explained and explicitly demonstrated on the test items in the post-scanning test.

Importantly, all learners reported that they consciously related and compared phonemes. Specifically, many learners reported that the structure of the last two consonants mirrored the sequence of the first two consonants in reversed order. Whenever learners stated explicitly that a specific phoneme was anticipated, we took this as evidence for a *forward prediction* strategy.

Using the *forward prediction strategy*, a learner would try to predict subsequent consonants during learning after hearing the first two consonants to find possible combinations. In the test phase, most of the learners realized that they could match the consonants in the inner pair during listening to the test items. They transformed the first consonant (e.g., “b” in “be-gi-ko-pu”) into the corresponding voiceless plosive (e.g.,“p”) and rehearsed the latter in working memory. During presentation of the fourth consonant, they checked if their expectations were met. For instance, if they were expecting a “p” during “be-gi-ko-pu,” the response would be “correct item”.

Using the *simple strategy*, a learner would try to keep the first two consonants in mind, and compare them with the following third and fourth consonant to find possible combinations. In the latest test phase after successful rule learning, a learner would keep only the first consonant in mind (e.g., “b” in “be-gi-ko-pu”) and compare it with the last one (“b” and “p” in “be-gi-ko-pu,” “b” and “t” in “be-gi-ko-tu”) for matching.

In summary, both strategies required speech stream segmentation and phoneme matching processes as well as phonological working memory. Indeed, in the structured interview, both groups, learners and non-learners, reported silent rehearsal of the syllables or phonemes. Specifically, after successful learning, learners used silent rehearsal to keep the consonants in memory for matching. Moreover, learners reported inner visualization of the syllables, vowels or consonants during learning and rule application in test phases. Accordingly, they visualized the consonants in the test phases that followed successful rule learning. Finally, non-learners reported difficulties in ignoring distracting information (e.g., vowels, which are irrelevant) during learning.

### fMRI Results

Since we were mainly interested in the difference of task-related activity in successful learners vs. non-learners, we investigated second-level contrasts directly addressing group comparison (i.e., learners > non-learners, and additionally non-learners > learners).

**FIGURE 4:**
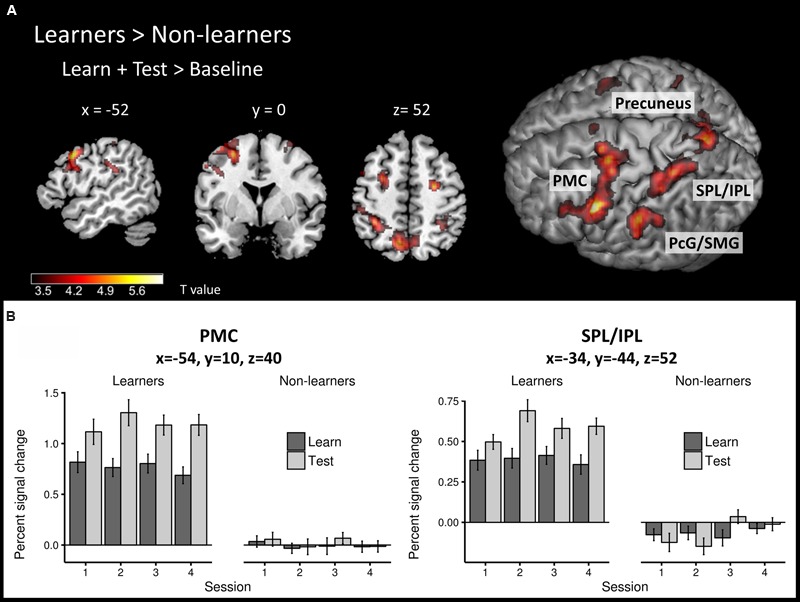
**Task-related neural activity across learning and test phases. (A)** Between-group comparison (learners > non-learners) for the learn+test > baseline contrast (two-sample *t*-test; FWE corrected, *p* < 0.05). **(B)** Percent signal change in the learn and test conditions across sessions at PMC and SPL/IPL peaks in each group. PMC – premotor cortex, SPL/IPL – superior parietal lobe/inferior parietal lobe, PcG – postcentral gyrus, SMG – supramarginal gyrus.

To identify brain regions that are involved in both successful learning and application of AG rules, we first compared the global learn + test > baseline contrast between learners and non-learners using a second-level, two-sample *t*-test. Overall, learners showed stronger activity in a large network of frontal and parietal areas (**Figure 4A**; **Table 2**) than non-learners.

**Table 2:** Local activation maxima in Montreal Neurological Institute (MNI) coordinates for the group comparison learners > non-learners.

Contrast	Location	Cluster size	Cluster FWE-corrected	p (unc)	*T*-value	*Z*-value	*x*	*y*	*z*
Learn + Test > Baseline	Left premotor cortex	1320	0	0	6.21	5.42	-54	10	40
					5.05	4.58	-26	0	56
					4.82	4.41	-26	6	70
	Right premotor cortex	275	0.032	0.004	5.09	4.61	26	-8	52
					3.76	3.54	30	2	68
	Left postcentral gyrus, supramarginal gyrus	345	0.014	0.002	5.08	4.61	-62	-20	36
					4.49	4.15	-66	-20	24
					4.22	3.93	-56	-34	26
	Left precuneus, superior/inferior parietal lobe, postcentral gyrus	1350	0	0	5.01	4.56	-8	-66	54
					4.79	4.38	-34	-44	52
					4.73	4.33	-42	-36	46
	Right postcentral gyrus, supramarginal gyrus	300	0.024	0.003	3.94	3.7	36	-32	44
					3.94	3.7	34	-48	48
					3.79	3.57	30	-44	56
Learn > Baseline	Left premotor cortex	272	0.032	0.004	5.12	4.64	-56	12	38
					4.53	4.18	-52	6	28
					3.56	3.38	-42	4	40
	Right superior/inferior parietal lobe	523	0.002	0	4.61	4.25	32	-48	48
					4.27	3.97	36	-48	58
					4.11	3.84	46	-40	44
	Left superior/inferior parietal lobe	414	0.005	0.001	4.12	3.85	-30	-48	44
					4.08	3.81	-42	-38	46
					3.91	3.67	-34	-58	60
Test > Baseline	Left postcentral gyrus, supramarginal gyrus	262	0.036	0.005	5.71	5.07	-62	-22	36
					3.67	3.46	-52	-34	26
	Left premotor cortex	1096	0	0	5.63	5.02	-54	10	40
					4.73	4.34	-24	4	70
					4.54	4.19	-26	0	54
	Left postcentral gyrus, supramarginal gyrus	281	0.028	0.004	4.73	4.34	-34	-44	52
					4.15	3.87	-44	-34	62
					4.13	3.85	-42	-36	44
	Left precuneus, superior/inferior parietal lobe	523	0.002	0	4.59	4.23	-8	-68	54
	Right cerebellum (lobule VI)	301	0.022	0.003	4.55	4.2	26	-64	-26
					4.22	3.93	30	-56	-32

The frontal cluster was located mainly in the left lateral PMC (peak at *x, y, z* = -54, 10, 40), partly extending to the middle, superior and inferior frontal gyri (pars triangularis and pars opercularis) as well as primary motor cortex. Increased neural activity was also found in the right PMC (peak at *x, y, z* = 26, -8, 52). The parietal clusters included significant activation in inferior and superior parietal lobes (IPL/SPL) bilaterally (peaks at *x, y, z* = -34, -44, 52; *x, y, z* = 34, -48, 48), left precuneus (peak at *x, y, z* = -8, -66, 54), bilateral postcentral gyrus (PcG) and SMG (peaks at *x, y, z* = -42, -36, 46; *x, y, z* = 36, -32, 44). Effect sizes for the peaks of the two major clusters in PMC and IPL/SPL in each run/group are summarized in **Figure 4B**.

**FIGURE 5:**
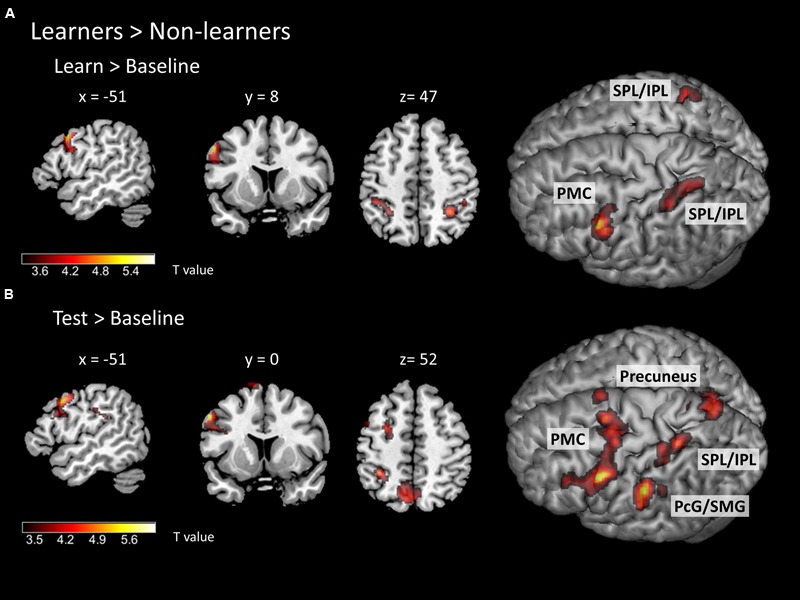
**Task-related neural activity during learning and test phases.** Between-group comparison (learners > non-learners) for **(A)** learn > baseline contrast and **(B)** test > baseline contrast (two-sample *t*-test; FWE corrected at a threshold of *p* < 0.05). PMC – premotor cortex, SPL/IPL – superior parietal lobe/inferior parietal lobe, PcG – postcentral gyrus, SMG – supramarginal gyrus.

To identify specific brain regions involved in either learning or application of the rule, we further computed contrasts for each condition (i.e., learn > baseline and test > baseline) between groups. In the learning > baseline contrast, learners displayed stronger activation in the left lateral PMC (i.e., at the border between left dorsal and ventral PMC; peak at *x, y, z* = -56, 12, 38) and bilateral IPL/SPL (peaks at *x, y, z* = -30, -48, 44; *x, y, z* = 32, -48, 48; **Figure 5A**; **Table 2**). In the test > baseline contrast, task-related activity also engaged left PMC (peak at *x, y, z* = -62, -22, 36) and left parietal areas, including precuneus (peak at *x, y, z* = -8, -68, 54), PcG and SMG (peak at *x, y, z* = -62, -22, 36), as well as IPL/SPL (peak at *x, y, z* = -34, -44, 52; **Figure 5B**; **Table 2**).

We did not find any significant activation in the second-level contrasts comparing non-learners vs. learners in either learn or test sessions, probably due to a strong variability of strategies in non-learners.

## Discussion

In this study, we investigated the functional underpinnings of successful learning of a complex phonological rule in an AG learning paradigm. Our main finding was that successful learning and rule application was associated with increased neural activity in a fronto-parietal network that encompassed left lateral premotor and prefrontal areas as well as bilateral regions in the IPL/SPL. These areas have been previously associated with learning and phonological processing (Tettamanti et al., 2002; Liebenthal et al., 2013). In the learning phase, the contribution of the IPL/SPL regions was more bilaterally distributed while the test phase showed a more left-lateralized parietal activation pattern. Notably, we did not find evidence for a contribution of left posterior IFG to AG learning. However, during rule application in the test phase, task-related premotor activity clearly extended into left IFG (i.e., pars opercularis and triangularis) and neighboring inferior frontal sulcus (IFS). This might indicate that the left IFG is not needed to support AG learning with our paradigm, but rather serves rule representation, as suggested by a previous AG learning study (Opitz and Friederici, 2004). That study used an AG that mimicked natural language rules and reported a shift of neural activation from the hippocampus in early learning stages to the posterior IFG when abstract rule representations were built. Our IFG/IFS cluster was located close to the activation pattern of another previous study that associated IFS activation with memory-related aspects during complex sentence processing (Makuuchi et al., 2009). This supports our claim of a key contribution of verbal working memory processes to our AG learning paradigm (see below).

In the following sections, we will discuss the cognitive processes associated with successful AG learning and the role of fronto-parietal regions in these processes. These data support our hypothesis that our task mainly required phonological processes and strongly engages phoneme comparison and verbal working memory.

### The Role of Phonological Processes in AG Learning: Contributions of Premotor Regions

With respect to the processes associated with phonological rule learning, our task required speech stream segmentation and phoneme comparison. It was previously argued that solving AG rules for syllable sequence structures can be done solely by phoneme matching, without the necessity to build hierarchies (Lobina, 2014).

Indeed, successful learners in our study reported the application of these strategies. Specifically, many of our successful learners relied on phoneme manipulation in the learning phase of our study (e.g., by transforming “b” into “p” in the forward prediction strategy described above). Other processes that might have been involved in learning our AG rule include discrimination of initial consonants of the syllables and phoneme monitoring. In accordance with our observation of increased task-related activation of lateral premotor areas (dorsal and ventral PMC) during successful AG learning, several previous neuroimaging studies demonstrated a contribution of the left PMC to speech segmentation and categorization tasks (Burton et al., 2000; Sato et al., 2009) as well as phoneme or syllable perception (Wilson et al., 2004; Pulvermueller et al., 2006) and production (Bohland and Guenther, 2006; Peeva et al., 2010; Hartwigsen et al., 2013).

A number of neuroimaging and non-invasive brain stimulation studies demonstrated that the left lateral ventral PMC is engaged in phoneme categorization (Burton et al., 2000; Wilson et al., 2004; Meister et al., 2007; Chang et al., 2011; Alho et al., 2012; Chevillet et al., 2013; Krieger-Redwood et al., 2013; Du et al., 2014). Burton et al. (2000) observed increased activity of left ventral PMC and adjacent inferior frontal gyrus during a phoneme discrimination task, when participants were asked to discriminate initial or final consonants in pairs of CVC syllables (e.g., “fat”–“tid,” “dip”–“ten”), compared to a pitch or a tone discrimination task. Moreover, Pulvermueller et al. (2006) showed a somatotopic activation in the left PMC during phoneme discrimination, for both subvocal production and passive listening tasks. The task specificity of this premotor activation was further shown by Krieger-Redwood et al. (2013), who used focal disruption of premotor activity induced by transcranial magnetic stimulation to demonstrate that the PMC is causally relevant for phoneme judgements, but not for speech comprehension. Together, the results of the previous and present studies suggest that the left lateral PMC is a key node for phoneme comparison, a process that is, among others, required during successful learning of speech sequences.

In a similar vein, another transcranial magnetic stimulation study by Sato et al. (2009) revealed a causal contribution of left PMC (overlapping our premotor cluster) to phonological segmentation. To successfully identify and apply the rule in our study, learners also used phonological segmentation to extract the consonants from a syllable sequence and compare the respective consonants on the corresponding positions. This process required basic acoustic analysis for phoneme identification, and verbal storage to discriminate phonemic contrasts when comparing syllables/consonants.

Premotor regions were also consistently associated with the planning and execution of speech gestures in previous neuroimaging studies (Gracco et al., 2005; Bohland and Guenther, 2006; Sörös et al., 2006). Accordingly, it was suggested that the recruitment of auditory-motor transformation and articulatory-based representations during phonological processing depends on the use of phonemic segmentation and working memory demands (Démonet et al., 1994; Zatorre et al., 1996; Hickok and Poeppel, 2004, 2007; Burton and Small, 2006). It was further argued that one specific strategy for performing short-term maintenance is phonological rehearsal by using inner speech (Herwig et al., 2003; see also Price, 2012 for review). This is well in line with the reported strategy from the successful learners in our study who used silent articulatory rehearsal of syllables or phonemes to compare specific consonants during learning. Note that to find the rule, our participants were specifically required to identify the pairing between voiced and unvoiced consonants according to the place of articulation.

Moreover, Chevillet et al. (2013) demonstrated that the left PMC (again overlapping with our cluster) was selectively activated during phoneme categorization but not acoustic phonetic tasks when subjects listened to a place-of-articulation continuum between the syllables “da” and “ga”. In that study, phoneme category selectivity in the PMC correlated with explicit phoneme categorization performance, suggesting that premotor recruitment accounted for performance on phoneme categorization tasks. This supports our hypothesis that successful learning requires distinguishing phonetic categories and matching corresponding phonemes.

### The Role of Phonological Processes in AG Learning: Contributions of Parietal Regions

Aside from the observed increases in premotor activity during successful learning and application of AG rules, we also found a strong upregulation of inferior and superior parietal areas (IPL/SPL) during both learning and test phase. Several previous studies have shown that left IPL regions and premotor areas jointly contribute to phonological tasks (Liebenthal et al., 2013), for example during the rehearsal of verbal sequences (Koelsch et al., 2009) and support sensorimotor integration (Poldrack et al., 1999; Buchsbaum et al., 2011; Liebenthal et al., 2013). The IPL was assigned a key role as an auditory-motor interface (Hickok and Poeppel, 2007, Rauschecker and Scott, 2009). Consistent with that view, left IPL activation was reported during phonemic categorization tasks, with the level of activity being related to the individual categorization ability (Jacquemot et al., 2003; Raizada and Poldrack, 2007; Desai et al., 2008; Liebenthal et al., 2013) and the learning of new phonemic categories (Kilian-Hütten et al., 2011). A meta-analysis by Turkeltaub and Branch Coslett (2010) also found significant activation likelihood in left IPL/SMG during categorical phoneme perception when subjects were required to focus on the differences between phoneme categories. This cluster overlaps with the location of the observed parietal activation in the learning phase in our study. These authors claimed that although the exact role of the IPL in categorical perception of speech sounds remains unclear, one explanation would be that increased IPL activity during phoneme perception might be related to phonological working memory processes (Baddeley, 2003a,b; Jacquemot and Scott, 2006; Buchsbaum and D’Esposito, 2008). As an alternative explanation, it was argued that the left IPL might serve as a sensorimotor sketchpad for distributing predictive information between motor and perceptual areas during speech perception and production (Rauschecker and Scott, 2009). The left IPL might also play a more domain general role in consolidating continuous features of percepts or concepts into categories and comparing stimuli during discrimination tasks (Turkeltaub and Branch Coslett, 2010).

Interestingly, the observed IPL/SPL activation in our study was more bilaterally distributed in the learning phase and more left-lateralized in the test phase. This might reflect different strategies or increased cognitive load during learning compared to application of the rule. Indeed, it was suggested that right hemispheric IPL/SPL regions might support their left-hemispheric homologs under demanding task conditions (Nakai and Sakai, 2014). Learning efficiency in second or artificial language learning paradigms was also associated with bilateral or right-hemispheric parietal networks (Kepinska et al., 2016; Prat et al., 2016). Moreover, bilateral contribution of parietal areas was associated with increased attention demands due to higher task difficulty during pseudoword vs. real word processing (Newman and Twieg, 2001), which might also have contributed to the observed bilateral parietal activation in the learning phase of our study.

Notably, in the test but not learning phase, additional left parietal activation was observed at the border between left postcentral and supramarginal gyrus as well as in the left precuneus. The left SMG has previously been associated with phonological working memory processes (Kirschen et al., 2006; Romero et al., 2006; Hartwigsen et al., 2010; Deschamps et al., 2014). This might indicate that during the test phase, our subjects relied more on phonological working memory processes to recall and apply the learned rule. Postcentral activation, on the other hand, was previously associated with motor-speech processes in tasks that required overt articulation (e.g., pseudoword repetition; Lotze et al., 2000; Dogil et al., 2002; Riecker et al., 2005; Peschke et al., 2009), or articulation without phonation (Pulvermueller et al., 2006). It was suggested that the left somatosensory cortex plays a crucial role in speech-motor control, forming part of a somatosensory feedback system (Guenther, 2006). Finally, left precuneus was associated with correct grammaticality decisions in another AG learning paradigm in a previous study (Skosnik et al., 2002) and might be related to retrieval success in working memory demanding tasks (Konishi et al., 2000; von Zerssen et al., 2001). Consequently, our observation of a strong upregulation of these regions in the test but not learning phase might indicate that subjects focused on phoneme manipulation in the learning, but relied stronger on working memory and rehearsal processes in the test phase. The upregulation of the precuneus might also point toward an engagement of mental imagery processes during the test phase since this region has been identified as a core node for visuo-spatial imagery previously (for review, Cavanna and Trimble, 2006). Moreover, activation of precuneus has been reported in previous fMRI studies on number comparisons or arithmetic calculations (Pinel et al., 2001; Nakai and Sakai, 2014).

We also found increased activity in the right cerebellum for learners vs. non-learners in the test phase. A contribution of the cerebellum is observed in many neuroimaging and electrophysiological studies in the cognitive and language domain (Marvel and Desmond, 2010; Price, 2012; De Smet et al., 2013). It was argued that the cerebellum plays an important role in the prediction of outcome associated with sensory input or actions (De Smet et al., 2013). Moreover, this area was also suggested to be involved in verbal working memory (Marvel and Desmond, 2010), potentially reflecting pre-articulatory or ‘internal speech’ processes. More specifically, the superior/lateral cerebellum (lobule VI) was associated with the encoding phase in covert speech and verbal working memory tasks. Together, the previous and present results indicate that the (right) cerebellum contributes to verbal working memory processes during speech processing that are necessary for successful encoding and rule application.

Overall, we found a contribution of fronto-parietal areas during both the learning and test phase in successful learners, indicating that a fronto-parietal network orchestrates successful AG processing. The stronger engagement of phonological processes in learners compared with non-learners in our study might reflect a more consistent application of the rule during and after successful learning. Particularly, non-learners might have lost attention or were engaged in wrong strategies such as focusing on intonation or rhythm, or simply relying on gut feeling or passive listening.

With respect to the interaction between both regions, it was previously argued that at least during verbal working memory tasks, left PMC provides phonological information to parietal areas (Herwig et al., 2003). Accordingly, these authors suggest that the concept of phonological storage can be regarded as a premotor-mediated top-down activation of internal representations of the memorized items in parietal regions for later recognition. However, it should be borne in mind that our task cannot solely be explained by verbal working memory processes, since it required explicit phonological manipulation (i.e., phonological segmentation and phoneme comparison). We argue that explicit phonological manipulation requires more than just temporary storing of the stimulus or its parts (constituent phonemes). Indeed, left IPL regions were previously associated with phonological manipulation processes. For instance, Peschke et al. (2012) reported increased IPL activity during phonological segmental manipulation as compared to prosodic manipulation. Moreover, it was suggested that activation of the premotor-parietal network during rehearsal tasks might represent formation and maintenance of sensorimotor codes that contributes not only to verbal processing, but also to a number of (motor) sequencing tasks (Jobard et al., 2003; Koelsch et al., 2009) and might thus indicate more domain general processing.

## Conclusion

In the present study, we investigated the functional underpinnings of successful learning and application of complex phonological rules, implemented in an AG learning paradigm with auditory presented structured syllable sequences. The observed fronto-parietal network comprised left premotor areas and bilateral superior/inferior parietal cortex. This network together with the reported strategies provides strong evidence for a core contribution of phonological processes, specifically phonological segmentation, phoneme comparison and inner rehearsal, as well as verbal working memory to successful AG learning. We might further speculate that successful AG learning might not require a specific contribution of the posterior IFG. However, this region comes into play during rule application in the test phase, further supporting the notion of a key role of the IFG in rule representation. Further studies might disentangle the exact role of the observed brain regions in AG learning and application, specifics of the time-course changes in activity and the interaction between these processes.

## Author Contributions

DG, JK, JM, and AF designed the experiment. DG and JK conducted the experiment. DG, JK, and GH performed the data analysis and interpretation. All authors contributed to the manuscript.

## Conflict of Interest Statement

The authors declare that the research was conducted in the absence of any commercial or financial relationships that could be construed as a potential conflict of interest.

## Footnotes

1 http://www.vislab.ucl.ac.uk/cogent2000.php

2 http://www.fil.ion.ucl.ac.uk/spm/

3 http://people.cas.sc.edu/rorden/mricron/index.html

## Supplementary Material

FIGURE S1 **Boxplots for behavioral performance (accuracy and reaction time) for learners and non-learners across sessions. (A)** Accuracy as percentage of correct answers across all trials in each session. **(B)** Reaction time across all correct trials in each session in milliseconds.
