# Supplementary figures and images for: Fronto-Parietal Contributions to Phonological Processes in Successful Artificial Grammar Learning
Source: Front Hum Neurosci. 2016 Nov 8;10:551. doi: 10.3389/fnhum.2016.00551 (PMC5100555; doi:10.3389/fnhum.2016.00551)

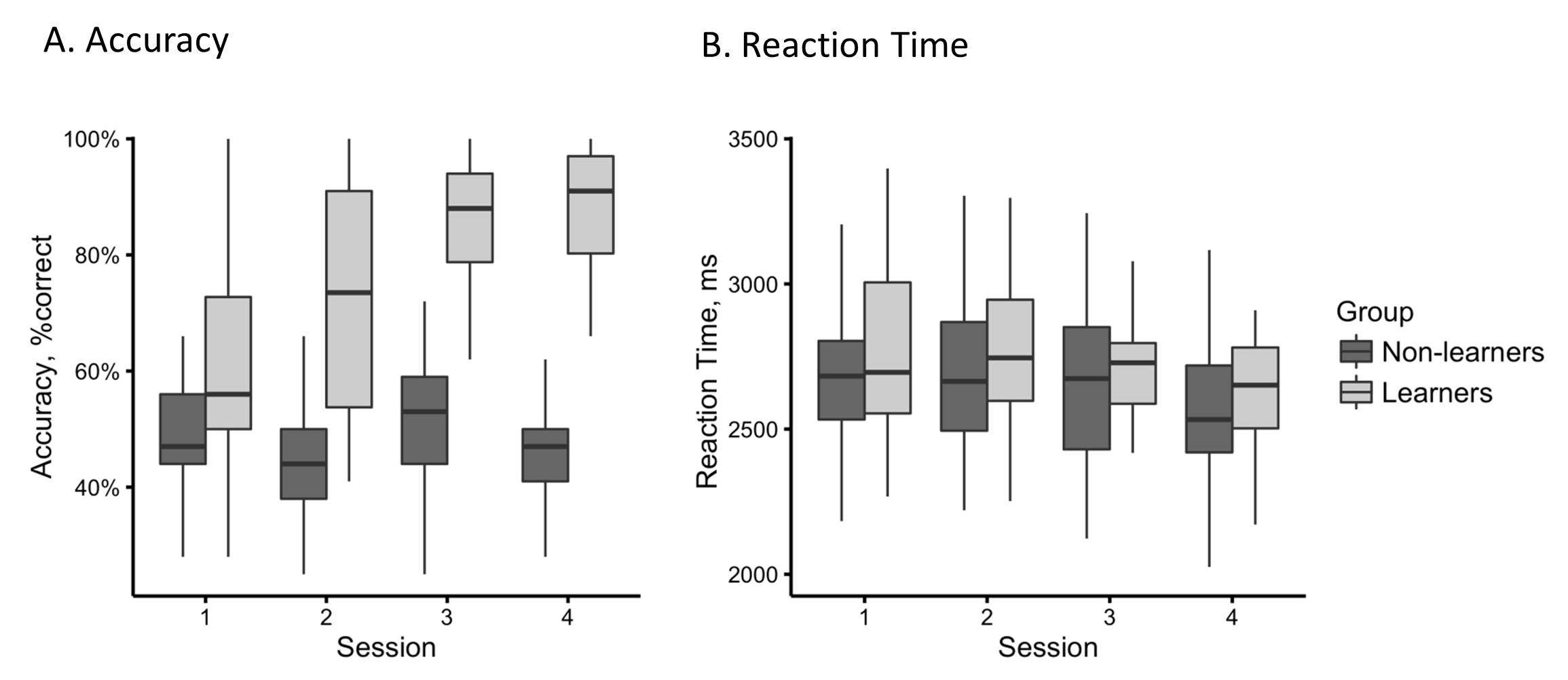

Supplement: FIGURE S1 — Boxplots for behavioral performance (accuracy and reaction time) for learners and non-learners across sessions. (A) Accuracy as percentage of correct answers across all trials in each session. (B) Reaction time across all correct trials in each session in milliseconds. [file Image_1.TIFF]
